# Evidence-based standardization of infectious disease biobanking: aligning specimen preservation and clinical metadata with *in vitro* diagnostic clinical trials requirements

**DOI:** 10.3389/fpubh.2026.1903960

**Published:** 2026-08-19

**Authors:** Chao Xia, Xingjian Liao, Sen Yang, Fang Cheng, Miaomiao Qi, Jing Ouyang, Bangyan Guan, Yemiao Chen

**Affiliations:** 1 Biobank, Chongqing Public Health Medical Center, Chongqing, China; 2 Clinical Research Center, Chongqing Public Health Medical Center, Chongqing, China; 3 Department of Nursing, Chongqing Public Health Medical Center, Chongqing, China

**Keywords:** biobanking, clinical metadata, *in vitro* diagnostic (IVD) clinical trials, infectious diseases, specimen preservation, standardization

## Abstract

**Background:**

Biobanks accelerate *in vitro* diagnostic (IVD) clinical trials by providing annotated, quality-controlled biospecimens. However, a persistent translational gap remains because the specific requirements of IVD trials—including sample matrices, preservation protocols, aliquot volumes, freeze–thaw tolerability, and clinical metadata—have not been systematically characterized. This study aims to establish an evidence-based framework to align infectious disease biobanking operations with regulatory-grade IVD trial demands by systematically characterizing the specimen preservation parameters and clinical metadata requirements of IVD trials.

**Methods:**

We retrospectively analyzed 140 infectious disease IVD trials (covering HIV/AIDS, COVID-19, viral hepatitis, and tuberculosis) conducted at a major Chinese infectious disease center between 2018 and 2025. Using a standardized extraction protocol, we systematically evaluated specimen preservation parameters (sample type, aliquot volume, storage temperature, duration, and freeze–thaw cycles), clinical metadata elements (universal and disease-specific), and control sample requirements.

**Results:**

Serum/plasma was the primary matrix (75.7% of trials), followed by whole blood (20.0%), sputum (13.6%), and swabs (10.7%). Predominant aliquot volumes were 0.5–1.0 mL for serum/plasma (52% of trials), 0.1–0.5 mL for whole blood (66%), and 2.0–3.0 mL for sputum (50%). Most trials preferred frozen specimens (48.6% exclusively frozen; 37.9% accepting both). For short-term preservation (2 °C–8 °C), a 7-day window was acceptable in 56% of serum/plasma protocols; for long-term preservation, −70 °C was required for 80% of serum/plasma and 100% of sputum samples. Strict freeze–thaw avoidance was mandated in 64% of serum/plasma, 72% of whole blood, and 60% of swab protocols. Core metadata (sample type and clinical diagnosis) were universally recorded (100%), whereas disease-specific laboratory parameters and control sample showed marked variation across disease categories.

**Conclusion:**

This single-center analysis offers preliminary evidence for a framework to standardize infectious disease biobanking practices tailored to IVD clinical trials. Implementation of the proposed preservation guidelines and tiered metadata framework, including standardized aliquot volumes, temperature-dependent storage protocols, strict freeze–thaw limitations, and strategic accrual of diverse control specimens, has the potential to optimize specimen utility, accelerate diagnostic development, and strengthen outbreak preparedness. However, external validation through multicenter studies is needed before these recommendations can be generalized to other settings.

## 1 Introduction

The persistent threat of emerging and re-emerging infectious diseases constitutes one of the most formidable challenges to global public health in the 21st century (1). The COVID-19 pandemic, which has claimed millions of lives and disrupted socioeconomic systems worldwide, serves as a stark reminder of the devastating impact of novel pathogens (2). Simultaneously, long-standing endemic diseases such as HIV/AIDS, tuberculosis (TB), and viral hepatitis continue to impose an enormous burden, particularly in low- and middle-income countries (3–5). The World Health Organization has repeatedly emphasized that rapid, accurate, and accessible etiological diagnosis is the cornerstone of effective epidemic control, guiding everything from individual patient management to public health decision-making and vaccine development. Consequently, there is an urgent and ongoing need to develop sensitive, specific, and field-deployable *in vitro* diagnostic (IVD) reagents that can perform reliably across diverse healthcare settings (6).

Before a novel diagnostic kit can be approved for routine clinical use, it must undergo rigorous, stepwise evaluation through IVD clinical trials—the regulatory prerequisite for market authorization enforced by agencies such as the National Medical Products Administration in China (7) and the Food and Drug Administration (FDA) in the United States (8). The World Health Organization (WHO) has established a comprehensive prequalification assessment procedure for IVDs, which requires manufacturers to submit clinical performance data generated through studies conducted using the specific specimen types claimed in the instructions for use (9). Similarly, the U.S. Food and Drug Administration (FDA) requires that clinical investigations of investigational *in vitro* diagnostic devices comply with the Investigational Device Exemption (IDE) regulations (10). The U.S. Centers for Disease Control and Prevention (CDC) has also emphasized the importance of well-characterized clinical specimens in diagnostic test development and validation, noting that the lack of such specimens is a critical bottleneck in the test development pipeline (11). These trials are designed to validate the analytical and clinical performance of the candidate kit, including its sensitivity, specificity, accuracy, and reproducibility. However, such trials are notoriously time-consuming and resource-intensive (11). A critical and often rate-limiting bottleneck is the collection of well-characterized, high-quality clinical specimens that meet strict inclusion and exclusion criteria. Recruiting eligible patients, obtaining informed consent, documenting detailed clinical histories, and procuring sufficient volumes of appropriate biospecimens—whether serum, plasma, whole blood, sputum, or swabs—frequently takes months or even years (12). This protracted timeline is particularly problematic during public health emergencies such as the COVID-19 pandemic, where delays in diagnostic validation can directly impede outbreak control efforts and cost lives.

Biobanks, as specialized platforms for the long-term preservation of human biospecimens and the systematic annotation of associated clinical and laboratory data, hold great promise to accelerate the IVD clinical trial pipeline. By providing ready-to-use, pre-collected, quality-controlled samples with comprehensive metadata, biobanks can bypass the lengthy *de novo* collection process. Regulatory authorities have recognized this potential; for instance, the National Medical Products Administration issued guidance in 2022 permitting the use of biobank-preserved human-origin samples for IVD reagent clinical trials (13). To ensure the quality and traceability of biospecimens, biobanks operate under Standard Operating Procedures (SOPs) that govern all aspects of specimen handling—from collection and processing to storage and distribution. These SOPs, informed by frameworks such as the ISBER Best Practices, are designed to standardize biobanking methods and minimize variation in biospecimen quality (14, 15). However, SOPs are often developed around a “storage-centric” paradigm, focusing on long-term preservation without a clear understanding of the specific downstream analytical requirements of IVD trials. As a result, despite regulatory endorsement, the actual translation of biobank resources into regulatory-grade evidence remains limited. The primary reason is that the specific requirements of IVD trials—regarding sample matrices, preservation protocols, aliquot volumes, storage stability, freeze–thaw tolerability, and associated clinical metadata—have not been systematically characterized. Consequently, many stored samples are either unsuitable for IVD use due to inappropriate processing (e.g., excessive freeze–thaw cycles, non-standardized aliquoting) or lack the essential clinical annotations required for trial eligibility.

To bridge this translational gap, it is essential to conduct an empirical, systematic analysis of real-world IVD clinical trial requirements across major infectious disease categories. In this study, we retrospectively analyzed 140 infectious disease IVD clinical trials conducted at a major infectious disease center in China between 2018 and 2025. Our objective was to characterize the biospecimen preservation parameters and clinical metadata requirements of these trials and to derive a standardized framework for infectious disease biobanking that can enhance specimen utilization for IVD clinical trials.

## 2 Methods

### 2.1 Study design and setting

This was a retrospective, descriptive study designed to systematically characterize the biospecimen preservation parameters and clinical metadata requirements of infectious disease IVD clinical trials. The study was conducted at the Chongqing Public Health Medical Center, a major tertiary referral hospital specializing in infectious diseases in Southwest China. All clinical trial projects reviewed were conducted at this center between January 1, 2018, and December 31, 2025.

### 2.2 Inclusion and exclusion criteria for clinical trial projects

Clinical trial projects were eligible for inclusion if they met all of the following criteria: (1) the trial evaluated IVD reagents for infectious disease detection; (2) the trial was approved and conducted at the Chongqing Public Health Medical Center; (3) the trial protocol explicitly specified at least one of the following biospecimen parameters: sample type, aliquot volume, storage temperature, storage duration, or maximum permissible freeze–thaw cycles; (4) the trial was completed or had reached the data lock point by December 31, 2025. Projects were excluded if they were: (1) non-diagnostic interventional drug trials without specified biospecimen handling protocols; (2) purely epidemiological surveys without defined laboratory specimen requirements; (3) preclinical or animal studies; or (4) trials with incomplete or missing preservation parameter documentation.

### 2.3 Data sources and trial identification

The clinical trial projects in this study were retrieved from the Clinical Trial Management Database of Chongqing Public Health Medical Center. This database archives all regulatory documents related to sponsor-initiated and investigator-initiated clinical trials approved by the Institutional Review Board of the hospital. A total of 153 infectious disease-related clinical trials were initially screened. After applying the inclusion and exclusion criteria, 140 trials were included for analysis. The main reasons for exclusion were incomplete retention of trial parameter data (3 trials) and non-diagnostic trial design (10 trials).

### 2.4 Data extraction and variable definitions

To assess inter-rater reliability, both reviewers independently extracted data from a random subset of 28 trials (20% of the total). Inter-rater agreement was calculated using Cohen’s kappa coefficient for categorical variables (sample type, storage temperature, freeze–thaw categories) and intraclass correlation coefficient (ICC) for continuous variables (aliquot volume, sample size). The kappa values ranged from 0.82 to 0.94 across the major categorical variables, indicating excellent agreement. The ICC for continuous variables was 0.91 (95% CI: 0.87–0.94). All disagreements were resolved by consensus or by a third reviewer.

#### 2.4.1 General trial characteristics

Disease category (HIV/AIDS, COVID-19, viral hepatitis, tuberculosis, or other), trial phase (if applicable).

#### 2.4.2 Biospecimen preservation parameters

*Sample type*: Classified as serum, plasma, whole blood, sputum, nasopharyngeal/oropharyngeal swab, urine, or other. Serum and plasma were combined for analysis given their functional equivalence in most diagnostic assays. Because a single trial could require multiple specimen types (e.g., serum/plasma, whole blood, and swabs simultaneously), each trial was counted in every applicable specimen category. Consequently, the sum of frequencies across specimen types exceeds the total number of trials (*n* = 140), and percentages for each specimen type were calculated using the total number of trials as the denominator (The raw data are presented in Supplementary Table S1).

*Aliquot volume*: The minimum volume of specimen required per test or per storage vial, recorded in milliliters (mL). Volumes were categorized into six groups to reflect their typical clinical applications and operational significance: ≤0.01 mL (ultra-micro assays, e.g., point-of-care testing), 0.01–0.1 mL (micro-volume molecular assays, e.g., PCR), 0.1–0.5 mL (routine molecular assays), 0.5–1.0 mL (standard immunoassays and serology—the most commonly required range for serum/plasma), 1.0–5.0 mL (multi-analyte profiling), and >5.0 mL (specialized assays requiring larger specimen volumes). A detailed interpretation of each volume category is provided in Table 1.

**Table 1:** Interpretation of sample aliquot volume categories.

Volume category	Typical application	Clinical/operational significance
≤0.01 mL	Ultra-micro assays (e.g., point-of-care, dried blood spots)	Minimal sample consumption; suitable for capillary blood
0.01–0.1 mL	Micro-volume assays (e.g., ELISA microplate, PCR)	Enables multiple tests per single collection
0.1–0.5 mL	Routine molecular assays	Most whole blood protocols fall in this range
0.5–1.0 mL	Standard immunoassays and serology	Modal volume for serum/plasma; balances single-assay sufficiency with minimizing waste
1.0–5.0 mL	Multi-analyte profiling	Required for comprehensive biomarker panels
>5.0 mL	Specialized assays (e.g., rare analyte detection)	Sputum protocols often require this volume

*Storage temperature*: Primary storage condition specified in the protocol, categorized as fresh (no defined temperature requirement, processed immediately), 2 °C–8 °C (refrigerated), −20 °C, or −70 °C (including −80 °C and liquid nitrogen).

*Storage duration*: Maximum recommended storage time at the specified temperature before analysis, categorized as <7 days, 7–14 days, 14–28 days, or long-term (≥28 days or indefinite).

*Freeze–thaw cycles*: Maximum number of freeze–thaw cycles permitted, recorded as 0 (avoidance), ≤1, ≤3, or ≤5.

#### 2.4.3 Clinical metadata

*Universal data elements*: Sample ID, sample type, collection date, visit date, patient ID, age, sex, clinical diagnosis, obstetric history.

*Disease-specific laboratory parameters*: For HIV/AIDS: genotype distribution, HIV viral load, TRFIA, Ct value; for COVID-19: Ct value, ECLIA; for viral hepatitis: genotype distribution, TRFIA, Ct value; for tuberculosis: sputum smear microscopy.

#### 2.4.4 Control sample requirements

Types of control specimens required for specificity or interference testing, categorized as healthy volunteers, non-target infectious disease patients (clinical mimics), autoimmune/rheumatic disease patients, pregnant women, or other.

### 2.5 Statistical analysis

Descriptive statistical methods were used exclusively, as the study aimed to characterize frequencies and distributions of specimen preservation parameters and clinical metadata elements without making inferential comparisons. Categorical variables (e.g., sample type, storage temperature, freeze–thaw categories) were summarized as counts and percentages. The denominator for percentage calculations was the total number of trials in which the specific parameter was clearly documented; missing data were reported separately. No imputation was performed for missing data; only available cases were analyzed. Given the descriptive nature of this study, no hypothesis-testing or inferential statistics (e.g., *p*-values) were applied.

### 2.6 Ethical approval

This study was a retrospective analysis of aggregated, de-identified data extracted from previously approved IVD clinical trial protocols. All original clinical trials had obtained ethics approval from the Institutional Review Board of Chongqing Public Health Medical Center and had collected informed consent from participants. For the present analysis, given the retrospective design and the use of de-identified data without any additional patient contact or intervention, the requirement for further informed consent was waived by the same Institutional Review Board. This waiver is consistent with Chinese regulations that permit exemption from informed consent for retrospective research using de-identified data (Measures for Ethical Review of Life Science and Medical Research Involving Humans, 2023, Article 32) (16).

## 3 Results

### 3.1 General characteristics of clinical trials and disease Spectrum

**Figure 1:**
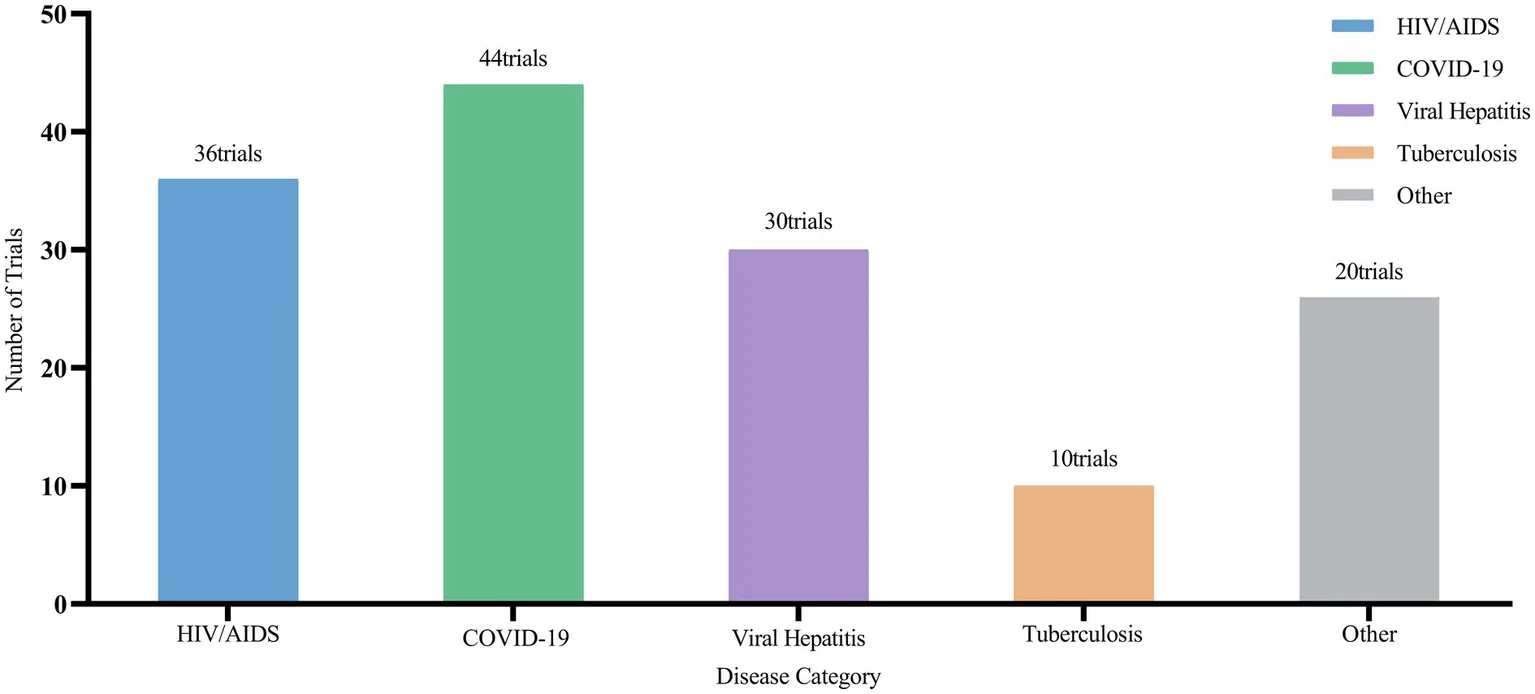
Distribution of disease types. The disease types include HIV/AIDS, COVID-19, viral hepatitis, tuberculosis, and others. Since some trials involved multiple disease types, each trial was classified into all corresponding categories. Accordingly, the total frequency of all categories exceeds the total number of trials (*n* = 140).

A total of 140 IVD clinical trials were included. Stratification by disease category revealed that COVID-19 (44 trials, 31.4%) and HIV/AIDS (36 trials, 25.7%) were the dominant focus areas, cumulatively accounting for over half of the portfolio. These were followed by viral hepatitis (30 trials, 21.4%) and tuberculosis (10 trials, 7.1%), with the remaining 20 trials (14.3%) encompassing other infectious etiologies (Figure 1). All 140 trials were multicenter in design, with Chongqing Public Health Medical Center serving as the one center for the trials included in this analysis. This disease distribution aligns closely with China’s current national public health priorities regarding major infectious disease control.

### 3.2 Predominance of serum and plasma matrices

**Figure 2:**
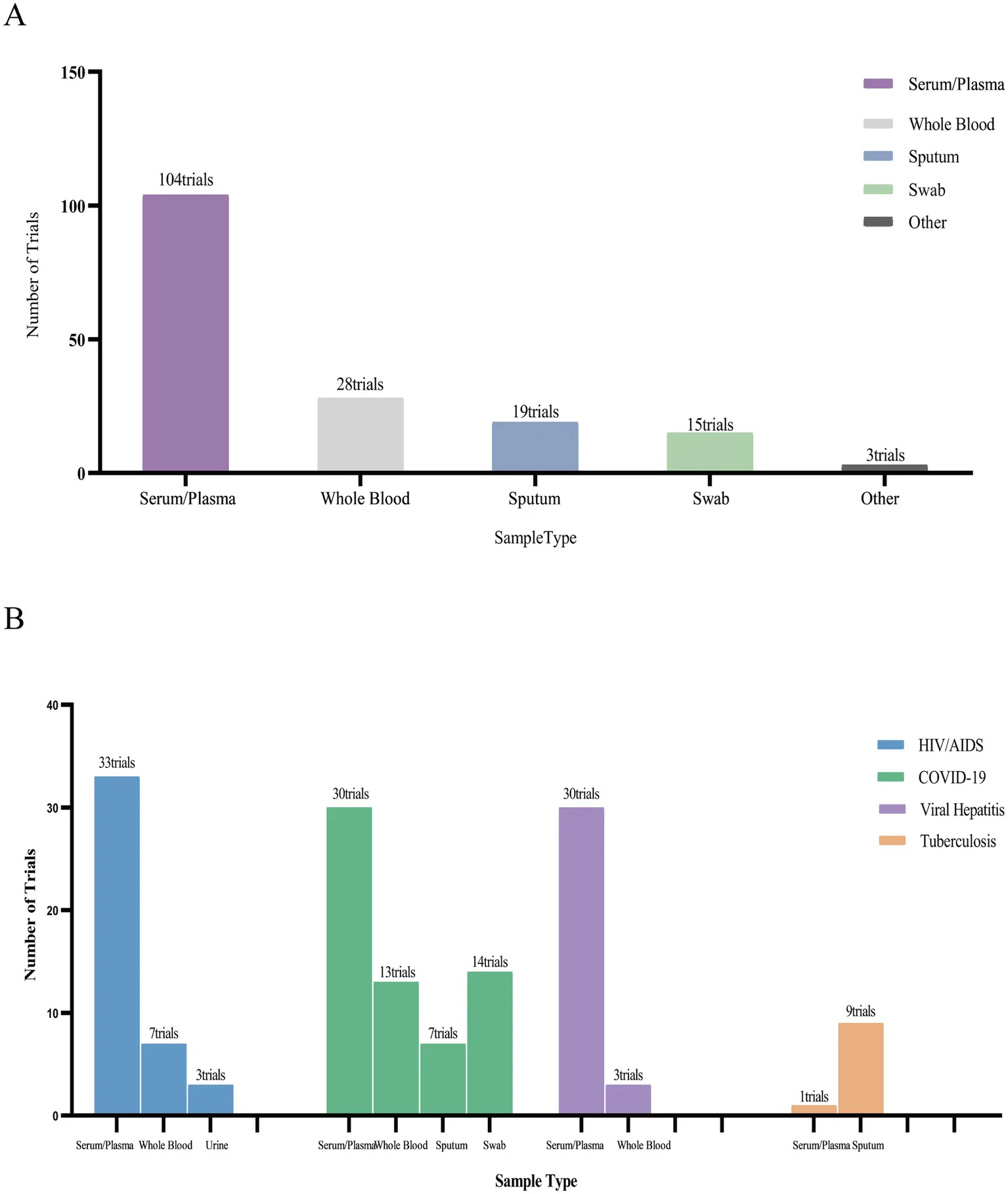
Specimen type distribution. **(A)** Specimen type frequencies across all trials. **(B)** Specimen type frequencies stratified by disease category (HIV/AIDS, COVID-19, viral hepatitis, tuberculosis). Because a single trial could permit multiple specimen types (e.g., serum, plasma, and whole blood simultaneously), each trial was counted in every applicable type. Consequently, the sum of frequencies across specimen types exceeds the total number of trials (*n* = 140).

As individual trials could require multiple specimen types, each trial was counted in every applicable specimen category. Serum/plasma was the predominant specimen matrix, required in 104 trials (74.3%). Whole blood ranked second with 28 trials (20.0%), followed by sputum (19 trials, 13.6%) and nasopharyngeal/oropharyngeal swabs (15 trials, 10.7%). Other sample types collectively accounted for the remaining 2.1% (Figure 2A).

When stratified by disease category, serum/plasma was required in 91.7% of HIV/AIDS trials and 100% of viral hepatitis trials. For COVID-19, serum/plasma remained most frequently required matrix (68.2%), followed by nasopharyngeal/oropharyngeal swabs (31.8%) and whole blood (29.5%). For tuberculosis, sputum was dominant specimen type (90%), with serum/plasma accounting for only 10% (Figure 2B). Given the small sample size, these findings should be interpreted with caution.

### 3.3 Heterogeneity in aliquot volume requirements

**Figure 3:**
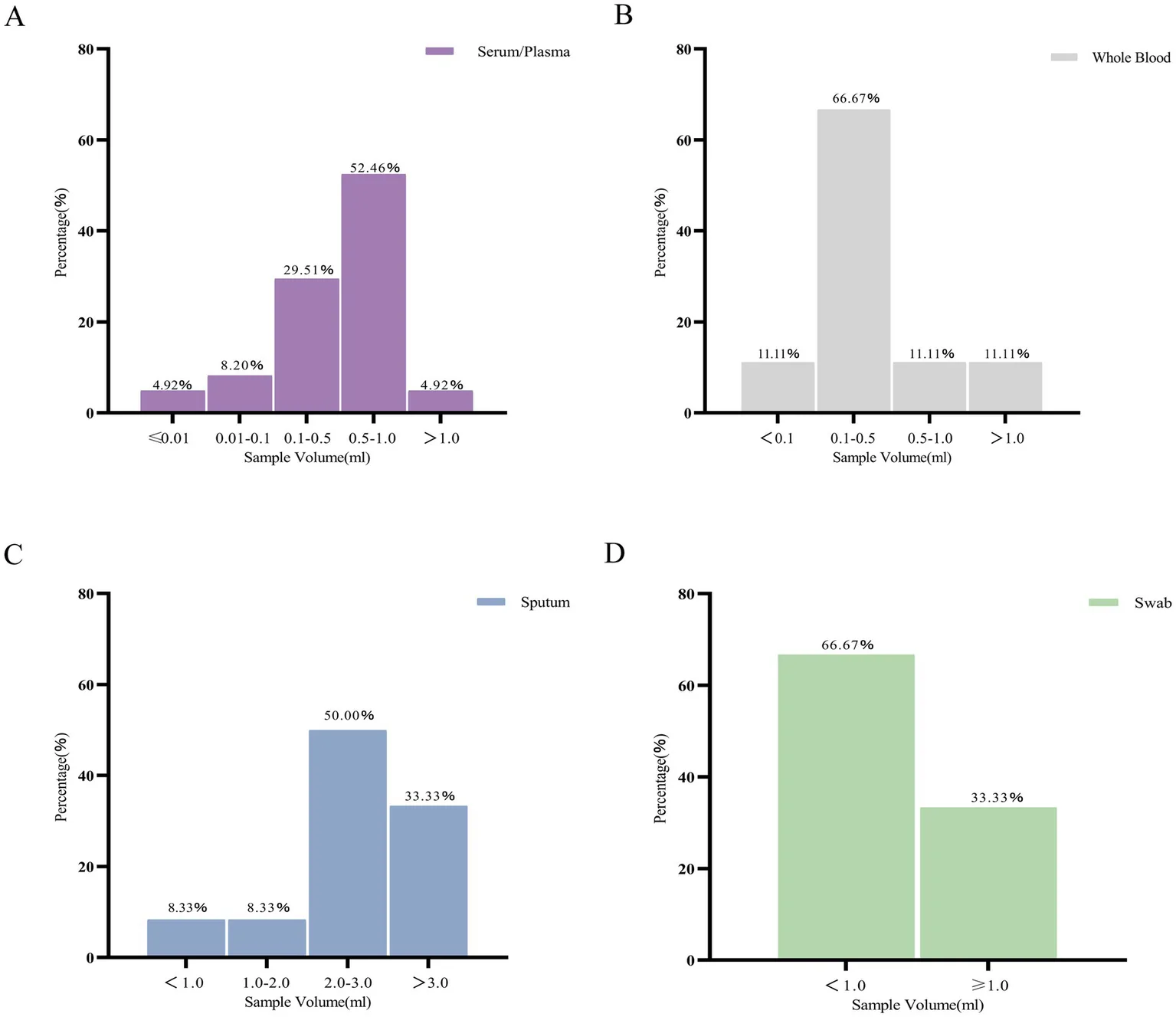
Distribution of aliquot volume requirements by specimen type. **(A)** Serum/plasma. **(B)** Whole blood. **(C)** Sputum. **(D)** Swabs. Each panel displays the percentage of trials requiring each volume category among protocols that explicitly specified aliquot volume requirements. Denominators: serum/plasma *n* = 61, whole blood *n* = 9, sputum *n* = 12, swabs *n* = 3. Note that a single trial could require multiple specimen types; therefore, the denominator for each panel is the number of trials in which that specimen type was required and the aliquot volume was clearly documented.

Aliquot volume requirements varied considerably among specimen types. When examined separately for each specimen type (Figures 3A–D), among protocols that clearly specified aliquot volumes, the most common requirement for serum/plasma was 0.5–1.0 mL (52%, 32/61) (Figure 3A), whereas whole blood protocols predominantly required 0.1–0.5 mL (66%, 6/9) (Figure 3B). Sputum specimens required larger volumes, with 50% (6/12) in the 2.0–3.0 mL range and 33% (4/12) exceeding 3.0 mL (Figure 3C). Swab specimens generally required small storage volumes, with 67% (2/3) below 1.0 mL (Figure 3D).

### 3.4 Temperature-dependent storage stability

**Figure 4:**
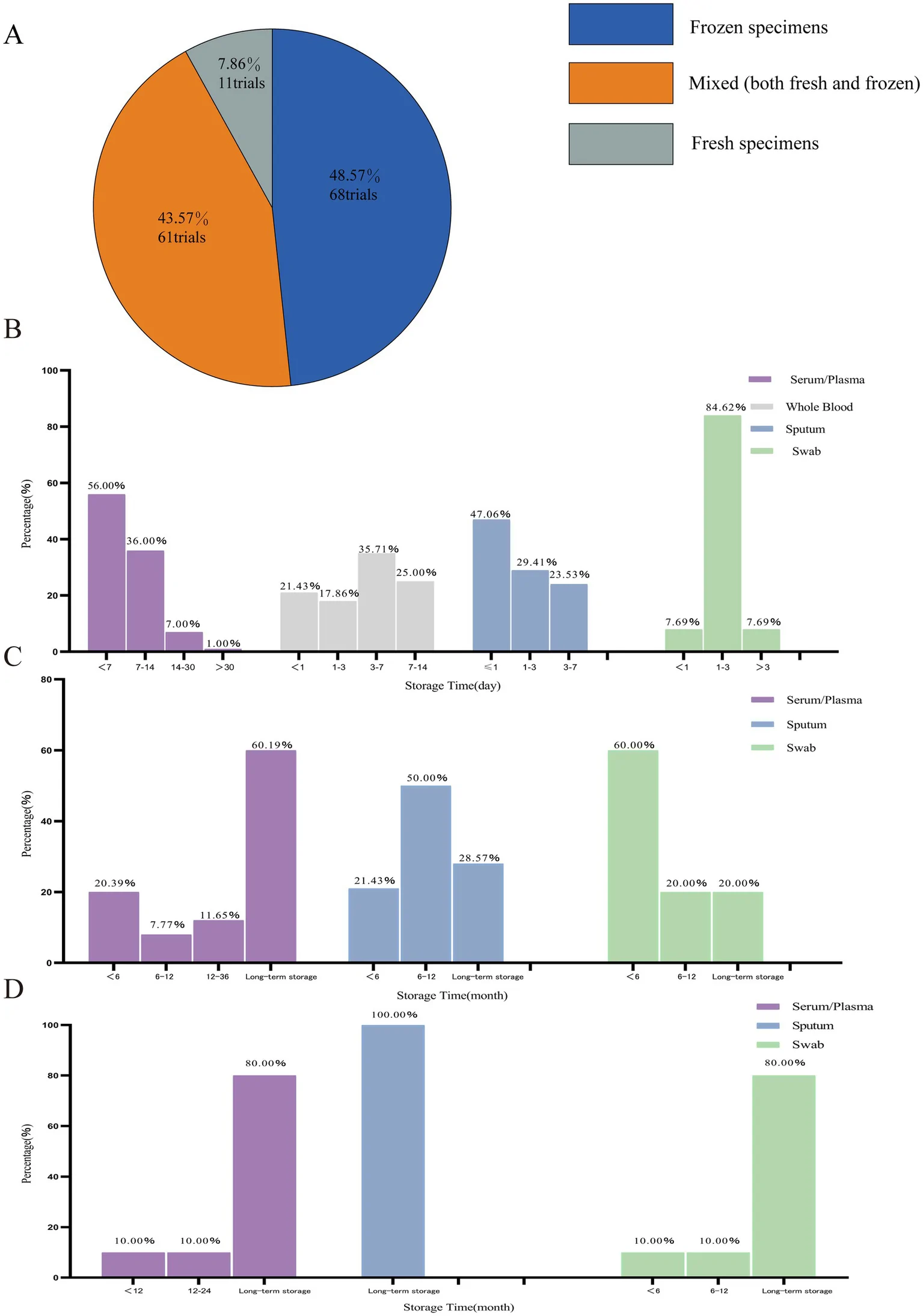
Specimen freshness status and storage durations at different temperatures. **(A)** Distribution of specimen freshness status. **(B)** Serum/plasma, whole blood, sputum, and swabs at 2 °C–8 °C. **(C)** Serum/plasma, sputum, and swabs at −20 °C. **(D)** Serum/plasma, sputum, and swabs at −70 °C.

Analysis of specimen freshness requirements revealed that frozen specimens were the most common category, with 68 trials (48.6%) requiring frozen samples exclusively. Fresh specimens alone were required in only 11 trials (7.9%). The remaining 61 trials (43.6%) accepted both frozen and fresh samples (Figure 4A). Storage protocols demonstrated a strong correlation between temperature conditions and intended storage duration. For short-term preservation (2 °C–8 °C), 56% of serum/plasma protocols specified a maximum duration of less than 7 days, while the allowable duration for whole blood showed a more scattered distribution (Figure 4B). For long-term cryopreservation, 60% of serum/plasma samples stored at −20 °C and 80% stored at −70 °C were designated for extended storage (Figure 4C). Notably, 100% of sputum samples and 80% of swab samples stored at −70 °C were categorized for long-term preservation (Figure 4D).

### 3.5 Freeze–thaw cycle restrictions

**Figure 5:**
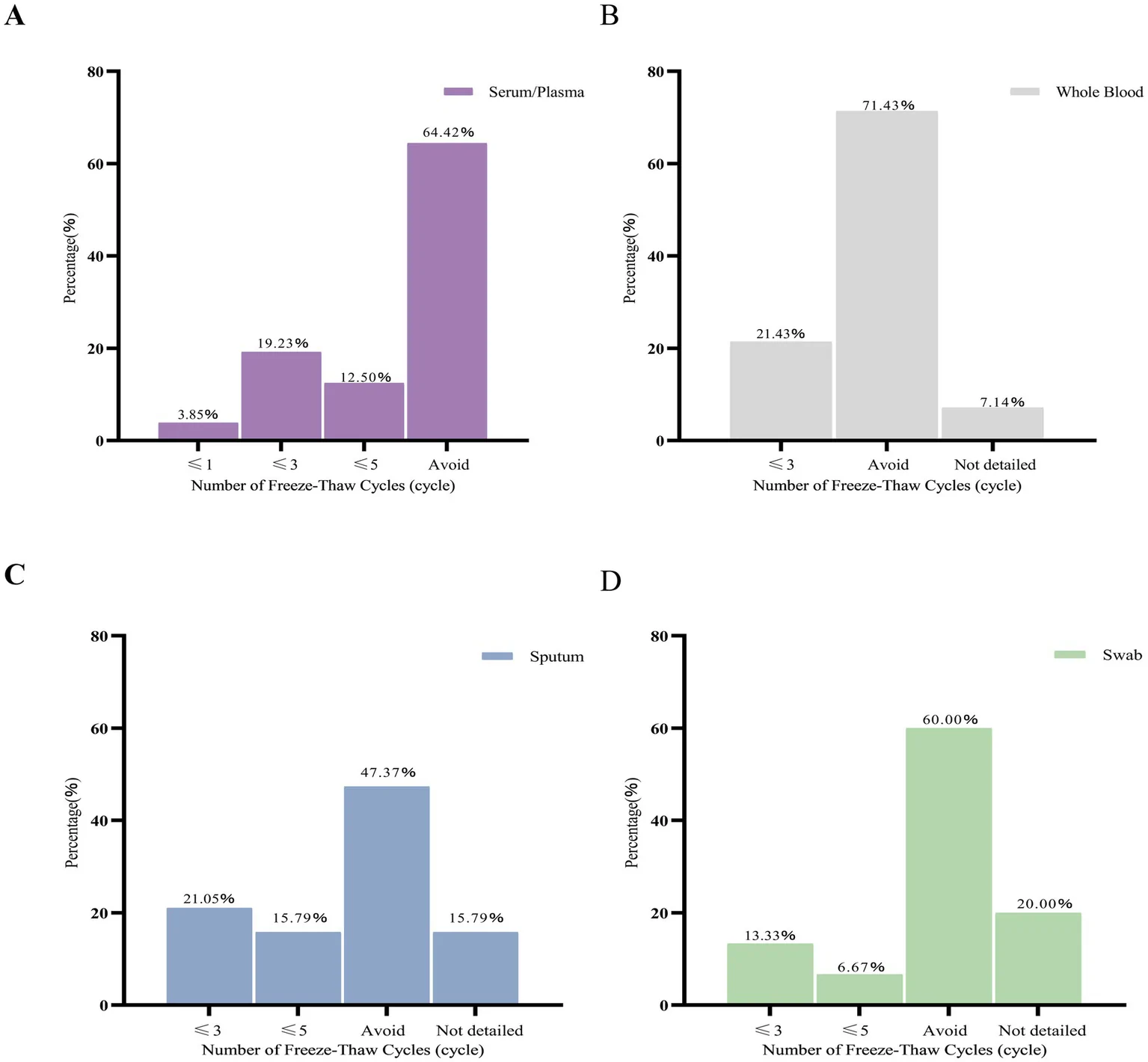
Distribution of permitted freeze–thaw cycles by specimen type. **(A)** Serum/plasma. **(B)** Whole blood. **(C)** Sputum. **(D)** Swabs. Each panel displays the maximum number of freeze–thaw cycles permitted in protocols specifying that specimen type. “Avoidance” indicates that no freeze–thaw cycle was permitted; “Not detailed” indicates that the protocol did not specify a limit.

Strict limitations on freeze–thaw cycles were observed across most specimen types. Complete avoidance of freeze–thaw cycles was the predominant requirement for whole blood (72%), serum/plasma (64%) and swab specimens (60%). Among protocols allowing freeze–thaw treatment, the threshold of no more than 3 cycles was the standard limit, applicable to 21% of whole blood and sputum specimens, 19% of serum/plasma specimens and 13% of swab specimens (Figures 5A–D).

### 3.6 Standardization of Core clinical metadata

**Figure 6:**
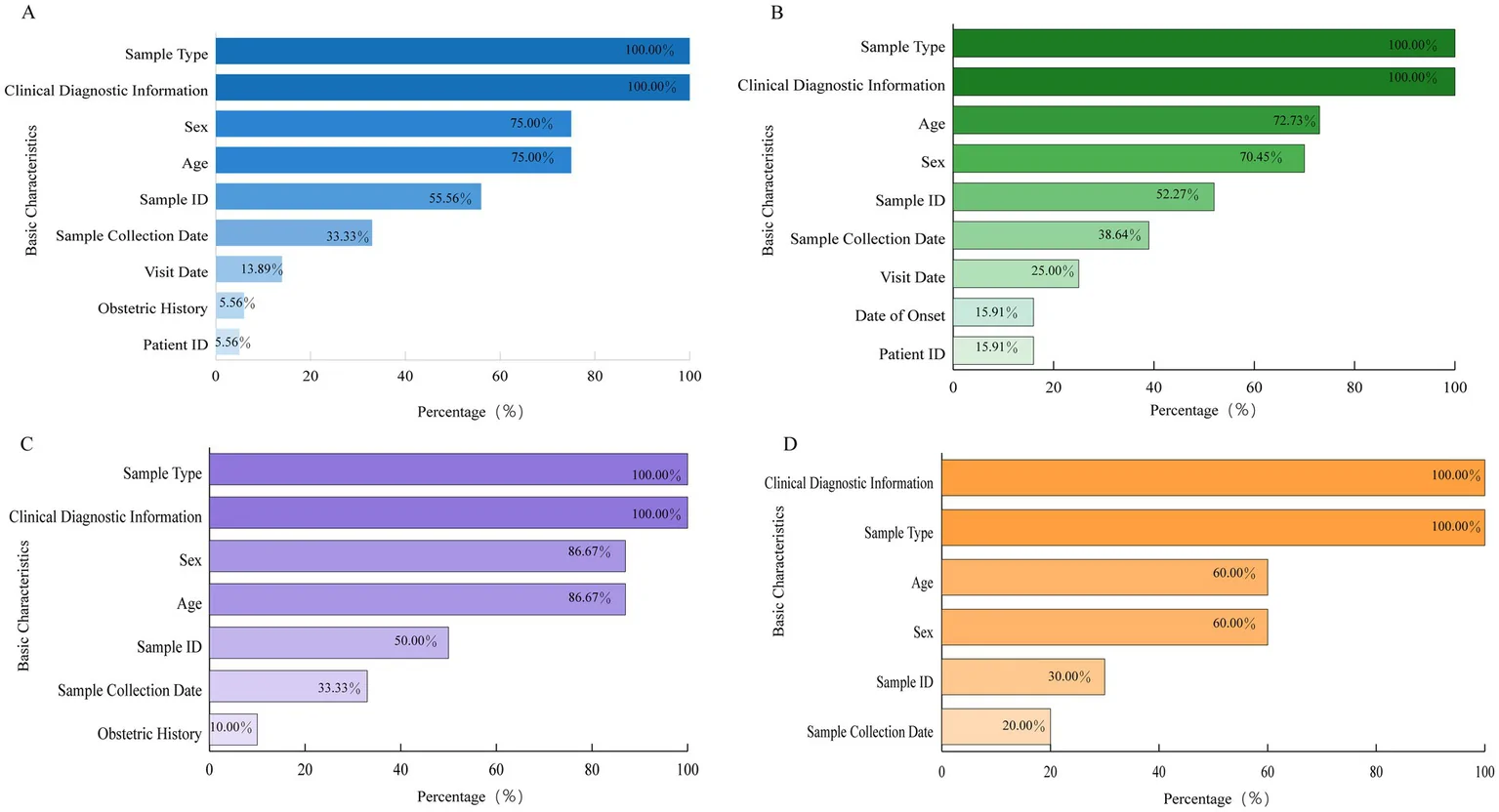
Distribution of core clinical metadata by disease category. **(A)** AIDS. **(B)** COVID-19. **(C)** Viral Hepatitis. **(D)** Tuberculosis.

Analysis of clinical data elements (CDEs) revealed that “Sample Type” and “Clinical Diagnosis” served as the core metadata, recorded in 100% of projects across all disease categories (Figures 6A–D). Demographic variables such as age and sex were the next most prevalent, with collection rates of 86.67% for viral hepatitis, 75% for HIV/AIDS, >70% for COVID-19, and approximately 60% for tuberculosis. Furthermore, traceability markers—specifically Sample ID and Collection Date—were emphasized across all cohorts, with Sample ID recording rates exceeding 40% in both HIV/AIDS and viral hepatitis projects (Figures 6A,C). Demographic variables such as age and sex were the next most prevalent, with collection rates of 86.67% for viral hepatitis, 75% for HIV/AIDS, >70% for COVID-19, and approximately 60% for tuberculosis.

### 3.7 Disease-specific laboratory biomarker profiles

**Figure 7:**
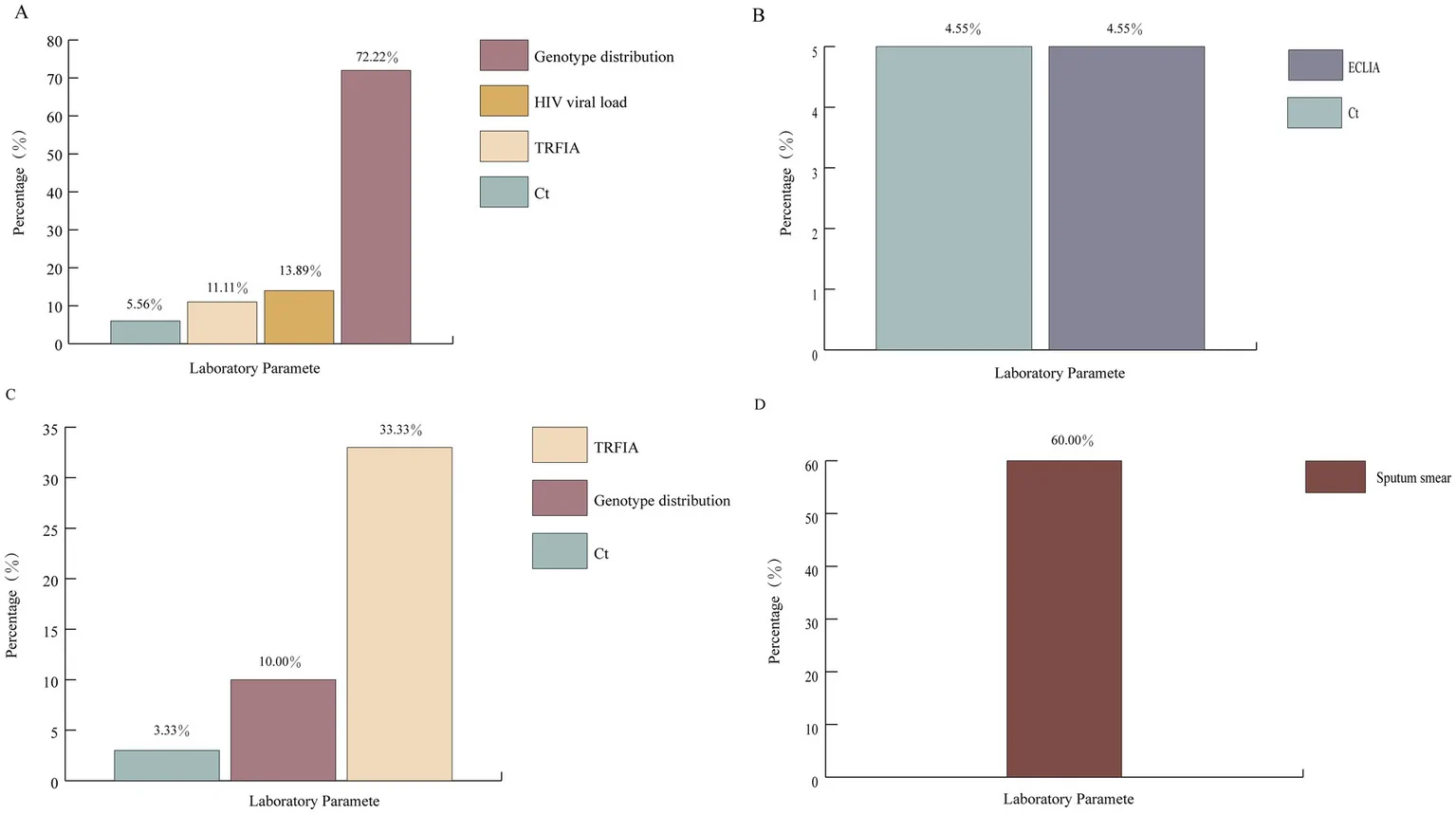
Distribution of laboratory parameters by disease category. **(A)** AIDS. **(B)** COVID-19. **(C)** Viral hepatitis. **(D)** Tuberculosis. TRFIA, time-resolved fluorescence immunoassay; ECLIA, electrochemiluminescence immunoassay.

The laboratory testing panels demonstrated high disease specificity. For HIV/AIDS (Figure 7A), the most frequently assessed biomarker was genotype distribution (72.22%), followed by HIV viral load (13.89%), time-resolved fluoroimmunoassay (TRFIA, 11.11%), and Ct value (5.56%). For COVID-19 (Figure 7B), the recorded parameters included Ct value (4.55%) and electrochemiluminescence immunoassay (ECLIA, 4.55%). For viral hepatitis (Figure 7C), TRFIA was the dominant parameter (33.33%), followed by genotype distribution (10.00%) and Ct value (3.33%). For tuberculosis (Figure 7D), 60.00% of protocols required sputum smear microscopy.

### 3.8 Variation in control sample requirements by disease etiology

Control group specifications varied considerably by disease etiology (Table 1). For COVID-19 IVD trials, control cohorts predominantly comprised patients with symptomatic respiratory infections caused by non-SARS-CoV-2 pathogens (72.7%) and healthy volunteers (50%). In HIV/AIDS projects, the primary requirement was for specificity controls derived from individuals infected with other viral pathogens (80.6%). Notably, for HIV-related immunological assays, there was substantial demand for samples from patients with autoimmune/rheumatic diseases (58.3%) and pregnant populations (41.7%) to assess potential interference. For liver disease assays, the requisite controls focused on excluding cross-reactivity, including patients with autoimmune disorders, rheumatoid factor positivity, or syphilis (90%), alongside healthy volunteers (66.7%). Tuberculosis trials exclusively required differential diagnosis controls from TB-negative patients presenting with upper respiratory tract infections, pneumonia, or tracheitis (100%) (see Table 2).

**Table 2:** Control group samples stratified by disease category.

Disease category	Control population	*N* (%)	Clinical/regulatory significance
COVID-19 (44)	Non-COVID-19 respiratory infection patients	32 (72.7%)	Assesses specificity against cross-reacting respiratory viruses
	Healthy volunteers	22 (50%)	Establishes reference intervals
HIV/AIDS (36)	Non-HIV viral infection patients	29 (80.6%)	Confirms specificity against related viruses
	Rheumatoid disease patients	21 (58.3%)	Evaluates interference from rheumatoid factor
	Pregnant women	15 (41.7%)	Assesses performance in special populations
	Healthy volunteers	11 (30.5%)	Establishes reference intervals
	Autoimmune disease patients	5 (13.9%)	Evaluates autoantibody cross-reactivity
Liver disease (30)	Autoimmune/rheumatoid factor-positive/syphilis patients	27 (90%)	Excludes cross-reactivity from interfering antibodies
	Healthy volunteers	20 (66.7%)	Establishes reference intervals
Tuberculosis (10)	TB-negative patients with respiratory infections	10 (100%)	Validates differential diagnosis from other pulmonary conditions

## 4 Discussion

This retrospective analysis of 140 infectious disease IVD clinical trials delineated the biospecimen and metadata requirements across four major disease categories. The key findings include the predominance of serum/plasma as the primary specimen matrix, a clear preference for standardized small-volume aliquots, stringent freeze–thaw restrictions, universal requirements for core clinical metadata, and considerable heterogeneity in control sample specifications.

The predominance of serum/plasma (75.7%) is consistent with the central role of serological and molecular assays in infectious disease diagnostics and aligns with collection priorities supported by the literature, which identifies blood as the most widely used specimen type due to its high viral load in systemic infections (17). However, the disease-specific variation observed in our study highlights that a “one-size-fits-all” approach to biobanking is inadequate. The reliance on sputum for tuberculosis (90% of TB trials) and swabs for COVID-19 (31.8%) reflects the tropism and transmission biology of these pathogens and is consistent with specimen prioritization in WHO diagnostic evaluation guidelines (18, 19). Crucially, our data quantify for the first time the extent of this variation across four major disease categories using real-world IVD trial protocols, providing biobanks with empirical evidence to inform diversified collection strategies. This finding suggests that biobanks serving IVD trials should maintain a diversified collection portfolio informed by local disease epidemiology.

Regarding aliquot volumes, the observed preference for serum/plasma aliquot volumes of 0.5–1.0 mL in our dataset is also consistent with clinical laboratory testing requirements. CDC test order documentation specifies that the minimum acceptable volume for serum is 0.5 mL (20), underscoring that 0.5–1.0 mL aliquots provide sufficient material for routine diagnostic assays. Further supporting this practice, the Canadian Partnership for Tomorrow’s Health reports that the median storage aliquot volume for both serum and plasma is 1.0 mL (21). In the study conducted by Parker et al. (22) blood specimens from participants were aliquoted at 0.5 mL for future research use. Notably, 48% of trials in our dataset required volumes outside this range, indicating that rigid standardization at a single volume may not accommodate all downstream applications. A practical approach would be to prepare multiple aliquots at the modal volume (0.5–1.0 mL for serum/plasma) while reserving a smaller number of alternative-volume aliquots where feasible.

The strong preference for frozen specimens (48.6% exclusively frozen, 43.6% accepting both) confirms that cryopreserved biobank inventories are broadly compatible with IVD trial requirements. These findings are favorable for biobank operations and indicate that most routinely stored biobank specimens can in principle be directly applied to *in vitro* diagnostic uses without additional preprocessing.

The 7-day acceptable window at 2 °C–8 °C for serum/plasma likely reflects a pragmatic compromise in multicenter trial logistics, including sample transport from remote sites, rather than an absolute stability threshold. A study by Lima et al. (23) found that when blood specimens were stored at 4 °C for 6 to 48 h, transcriptome sequencing revealed only a small number of differentially expressed genes, indicating that short-term storage has a minimal impact on specimen quality. Another study demonstrated that COVID-19 plasma samples retained nearly all valid information even after storage at 4 °C for up to 7 days (24). However, another published stability studies for labile analytes such as cytokines and coagulation factors generally recommend shorter holding times (≤48 h) at refrigerated temperatures (25, 26). Biobanks should interpret this finding conservatively: while 7 days may be acceptable for stable analytes targeted by routine IVD assays, specimens intended for more sensitive molecular or immunological analyses may require more stringent cold-chain protocols. In protocols for long-term biobank storage, 80% of serum/plasma specimens and 100% of sputum specimens require storage at −70 °C. This requirement is consistent with the recommendations for molecular specimens specified in pre-analytical guidelines (27, 28).

Our results suggest that freeze–thaw of specimens should be avoided as far as possible in IVD trials, with complete prohibition specified in 64%–72% of trial protocols, and freeze–thaw cycles should be limited to fewer than three if unavoidable, which is consistent with existing consensus. A systematic review encompassing 46 biobank-related studies on serum and plasma specimens reported that freeze–thaw cycles impair specimen stability in a dose-dependent manner. More than 10 freeze–thaw cycles lead to severe sample degradation with alterations in 70% of biomarkers and widespread damage to various enzymes; notably, even ≤5 cycles can significantly alter enzymatic activities and affect 43% of tested enzymes (29). More conservative thresholds are typically applied in IVD trial protocols, likely reflecting regulatory caution. This finding has direct operational implications: specimens should be dispensed into the minimum necessary volume at initial processing, and each aliquot should ideally be thawed only once.

While general metadata standards such as MIABIS and BRISQ provide foundational guidance for biobank sample annotation (30, 31), to our knowledge, no framework has been empirically derived specifically for infectious disease IVD trials. Based on our findings, we propose a tiered metadata framework tailored to this context: Tier 1 (mandatory) includes Sample ID, Sample Type, Collection Date, Clinical Diagnosis, Age, and Sex—elements that were universally recorded (100%) across all disease categories in our dataset, confirming their essential status. Tier 2 (strongly recommended) includes key disease-specific laboratory results (e.g., genotype distribution for HIV, TRFIA for hepatitis, sputum smear for TB)—parameters identified empirically from our disease-stratified analysis as the most relevant contextual information for matching samples to appropriate downstream assays. Tier 3 (optional but valuable) includes detailed clinical annotations such as obstetric history and symptom onset date—data that, while not universally required, provide valuable context for specific research questions. Unlike generic standards, our framework is anchored in real-world IVD trial requirements and directly addresses the operational needs of biobanks serving diagnostic development, thereby bridging the gap between general biobanking guidance and the specific demands of regulatory-grade IVD trials.

The heterogeneity in control sample requirements represents a frequently overlooked challenge for biobanks. Most biobanking efforts focus on accruing “positive” cases, yet our data indicate that IVD trials consistently require diverse control populations including clinical mimics (patients with similar symptoms but different etiologies), interference populations (autoimmune disease, pregnancy, rheumatoid factor positivity), and healthy volunteers. This aligns with WHO prequalification requirements, which mandate cross-reactivity studies using specimens from non-target infections (e.g., HBV/HCV, CMV, EBV, HTLV, malaria, tuberculosis), interference studies assessing endogenous substances including autoimmune conditions, and clinical performance evaluation across all claimed specimen types (9). These requirements collectively underscore the critical importance of analytical specificity in IVD performance assessment. The pattern of requirements suggests that biobanks should adopt a proactive negative library strategy—systematically collecting and annotating control specimens with the same rigor applied to case samples.

Several limitations should be acknowledged. First, this was a single-center retrospective analysis based on data from IVD trials conducted at one specialized infectious disease hospital in China that served as the one center for these multicenter studies; the specimen requirements observed may not be fully generalizable to trials conducted under other regulatory frameworks or in other geographic settings. Second, the relatively small number of tuberculosis trials (*n* = 10) limits the robustness of conclusions specific to this disease category; these findings should be considered exploratory and hypothesis-generating rather than definitive. Third, our analysis characterized the stated requirements in trial protocols but did not assess actual specimen utilization rates or trial outcomes; the extent to which adherence to these parameters affects trial success remains to be determined. Future multicenter studies incorporating trial outcome data would strengthen the evidence base.

## 5 Conclusion

In conclusion, this single-center retrospective analysis offers several contributions to the field of infectious disease biobanking. First, to our knowledge, this is the first systematic, empirical characterization of IVD trial specimen requirements across four major infectious disease categories (HIV/AIDS, COVID-19, viral hepatitis, and tuberculosis) based on real-world data from 140 trials. Second, we have quantified the specific aliquot volume preferences, temperature-dependent storage protocols, and freeze–thaw restrictions that are actually specified in IVD trial protocols, providing an evidence base that has been lacking for biobank operational decisions. Third, we propose a tiered clinical metadata framework tailored to IVD trials that balances comprehensiveness with feasibility, addressing a gap between current biobank practices and the FAIR principles. Fourth, we have identified and quantified the substantial and previously underappreciated heterogeneity in control sample requirements across disease categories, providing empirical justification for a strategic “negative library” approach. Implementing the proposed preservation guidelines and metadata frameworks, including standardized aliquot volumes, temperature-dependent storage protocols, strict freeze–thaw limitations, and strategic accrual of diverse control specimens, can optimize specimen utility, accelerate diagnostic development, and strengthen outbreak preparedness. However, given the single-center, retrospective nature of our analysis, these findings should be considered preliminary and hypothesis-generating. Validation through multicenter, prospective studies incorporating trial outcome data would be a valuable next step before broader adoption of these recommendations.

## Data Availability

The raw data supporting the conclusions of this article will be made available by the authors, without undue reservation.
